# ‘Can do’ versus ‘Do do’ in nursing home residents: identification of contextual factors discriminating groups with aligned or misaligned physical activity and physical capacity

**DOI:** 10.1186/s11556-024-00365-4

**Published:** 2024-11-12

**Authors:** Michael Adams, Alexander Elser, Madeleine Fricke, Lydia Jaufmann, Bettina Wollesen, Thomas Muehlbauer, Carl-Philipp Jansen, Michael Schwenk

**Affiliations:** 1 Institute of Sports and Sports Sciences, Heidelberg University, 69120 Heidelberg, Germany; 2 Department of Social Work and Health, University of Applied Sciences and Arts, 31134 Hildesheim, Germany; 3 Department of Biological Psychology and Neuroergonomics, Technical University of Berlin, 10623 Berlin, Germany; 4 Department of Human Movement Science, University of Hamburg, 20148 Hamburg, Germany; 5 Division of Movement and Training Sciences, Biomechanics of Sport, University of Duisburg- Essen, 45141 Essen, Germany; 6 Department of Clinical Gerontology and Geriatric Rehabilitation, Robert Bosch Hospital, 70376 Stuttgart, Germany; 7 Geriatric Center, Heidelberg University Clinic, Heidelberg University, 69126 Heidelberg, Germany; 8 Human Performance Research Centre, Department of Sport Science, University of Konstanz, 78464 Konstanz, Germany

**Keywords:** Nursing home, Long-term care, Physical activity, Physical capacity, Life-space mobility, Activities of daily living

## Abstract

**Background:**

Physical activity (PA) is fundamental to nursing home residents’ health. Likewise, physical capacity (PC) is essential to carry out activities of daily living. Although PC and PA are associated, misalignment has been reported in specific subgroups. Increased PC is oftentimes not linked to high PA (i.e., Can do - don’t do) and vice versa (i.e., Can’t do - do do). Therefore, identifying other contextual factors influencing PA in misaligned groups is important. This study aimed to identify contextual factors in nursing home residents with aligned or misaligned PA and PC.

**Methods:**

In total, 180 nursing home residents (≥ 65 years, 79.4% females) were divided into four quadrants (Q1: Can do - do do; Q2: Can do - don’t do; Q3: Can’t do - do do; Q4: Can’t do - don’t do) based on thresholds for PA (≥ or < 2,500 steps/day) and PC (≤ or > 0.5 m/s gait speed). Kruskal-Wallis H test and effect sizes (ES) were applied to analyze quadrants’ differences regarding PA (steps per day), objective motor capacity, life-space mobility, activities of daily living (ADL), psychosocial well-being, cognition, subjective mobility-related concerns, and spatial orientation.

**Results:**

Specific contextual factors differed significantly between the groups. Compared to Q1, Q2 presents a significantly lower life-space mobility (ES: 0.35) and objective motor capacity (ES: 0-36-0.49); Q3 has a lower objective motor capacity (ES: 0.55–1.10); Q4 shows lower independence in ADL (ES: 0.57), life-space mobility (ES: 0.48), subjective mobility-related concerns (ES: 0.38) and objective motor capacity (ES: 0.99–1.08). No significant group differences were found for psychosocial well-being, cognition, and spatial orientation.

**Conclusions:**

This study provides new insights into PA behavior of nursing home residents. Key variables linked to PA are objective motor capacity, life-space mobility, ADL, and subjective mobility-related concerns. Surprisingly, some potentially impactful variables such as cognition, orientation, and psychosocial well-being did not differ between the groups. This may suggest that these variables may not represent key targets for interventions aiming to improve PA. This study builds the foundation for further research into the underlying mechanisms behind PA behaviors and supports future efforts to plan specific, targeted interventions for nursing home residents.

**Trial registration:**

The trial was prospectively registered at DRKS.de with registration number DRKS00021423 on April 16, 2020.

**Supplementary Information:**

The online version contains supplementary material available at 10.1186/s11556-024-00365-4.

## Background

Nursing home residents show dramatically low levels of physical activity (PA) [1] far below the World Health Organization’s recommended threshold of at least 150 min per week of moderate-intensity aerobic physical activity or 75 min per week of vigorous-intensity aerobic physical activity [2]. These are negatively associated with multiple health-related factors including quality of life, depression [3, 4], functional mobility, and well-being [5].

Besides PA, physical capacity (PC), defined as what a person can do in a standardized environment [6], is also low in nursing home residents [7]. An important marker for PC, which is highly relevant for everyday function, is gait speed. This is recommended and often used to measure PC in older people [6]. Low levels of PC, like a low gait speed, lead to a 2.58-fold increased risk of dying within five years [8] and a lower quality of life [9].

Given the high relevance of PA and PC, improving both components is an important target for nursing home residents. A common approach is training PC, assuming that this will result in greater PA. However, research shows that PC often has only small associations with PA, leaving discrepancies between measured PC and PA levels [10–13]. These discrepancies apparently are linked to multiple determinants of nursing homes residents’ PA other than PC.

In older adults in general, important variables influencing PA are aspects of life-space mobility, activities of daily living (ADL), psychosocial well-being, emotional status, subjective mobility-related concerns as well as the PC. More precisely, life space mobility is closely related to PA [14] and helps to express the quality of PA due to the integration of social participation in nursing home residents [15]. The navigation through different life-spaces requires spatial orientation processes, e.g., remembrance of target localization, awareness of distance and directions [16, 17]. Looking at ADL, it is notable that PA influences them positively, allowing older adults to maintain functional independence for a longer time-span [18]. Previous studies have also shown a positive association between PA levels and psychosocial wellbeing in older adults [3, 4]. Changes in emotional states can result in a reduced self-esteem in older adults [19] which in turn can reduce the level of PA. One reason for this may be that the emotional state triggers subjective mobility- and fall-related concerns in nursing home residents [20]. Hence, the interaction of several variables can influence PA and lead to discrepancies between PC and PA in nursing home residents. In order to better shape PA-enhancing interventions in this population, it is important to determine the impact of those variables.

The PC–PA quadrant or ‘Can do – do do’ concept is one method to better understand the discrepancies between PC and PA [21], dividing people into four different quadrants. Two axes are formed by the PCs and PA, respectively, that distinguish the two categories ‘can do’ and ‘can’t do’ using defined thresholds. People with high PC are in the ‘can do’, those with low PC in the ‘can’t do’ group. PA-wise, those who are active will be located in the ‘do do’ category; those who are inactive will be situated in the ‘do not do’ category. In this way four quadrants are created that make up the concept: (Q1) ‘Can do – do do’ (high PC, high PA), (Q2) ‘Can do – don’t do’ (high PC, low PA), (Q3) ‘Can’t do – do do’ (low PC, high PA), and (Q4) ‘Can’t do – don’t do’ (low PC, low PA). While Q1 and Q4 represent a plausible, aligned ratio of PC to PA, the ratio of PC to PA in Q2 and Q3 is misaligned. The concept has already been applied by Koolen et al. [21] in people with chronic obstructive pulmonary disease and in our group to analyze PA patterns in older people [22]. Here, we apply the concept to gain more insight into the relationship between PC and PA and related factors in nursing home residents. Especially in nursing home residents with misaligned PC and PA (quadrants [2] and [3] described above), this could help to align capacity with activity behavior, and improve health conditions.

Thus, the purpose of this study is to use the PC-PA quadrant concept to identify subgroups of nursing home residents that may help explain the misalignment between their PC and PA. It aims to (1) determine the distribution of nursing home residents across the proposed PC-PA quadrant concept, and (2) explore whether and to what extent there are differences in contextual factors between nursing home residents classified into misaligned vs. aligned PC-PA quadrants.

1. We hypothesized that the aligned group Q1 would perform significantly better than the aligned group Q4 on all dependent variables, including objective motor capacity, life-space mobility, ADL, psychosocial well-being, cognition, subjective mobility-related concerns and spatial orientation.
2. We hypothesized to find significant differences between the aligned groups compared to misaligned groups with similar PC but different PA (Q1 vs. Q2; Q3 vs. Q4) on specific dependent variables including life-space mobility, ADL, psychosocial well-being, cognition, subjective mobility-related concerns and spatial orientation.

## Methods

### Study design

In this cross-sectional study we used preliminary baseline data from an intervention study [23] that took place between 2020 and 2022 in a total of 17 nursing homes spread over three German metropolitan regions (Berlin, Rhine-Neckar Metropolitan Region, around Duisburg and Essen). All participants provided written informed consent. The ethics committee of the TU Berlin, Germany has approved the study protocol (No GR_14_20191217) and the trial was registered at Deutsches Register Klinischer Studien (DRKS) with registration number DRKS00021423. Further information on trial design and ethics can be found in the study protocol [23].

### Inclusion and exclusion criteria

Based on the inclusion criteria, suitable nursing home residents were preselected by staff and then verified by the researcher who approached them to participate in the study. The inclusion criteria encompassed (1) willingness to participate (2), capacity to engage in group activities, (3) ability to walk (with or without walking aid), and (4) the ability to understand and execute simple instructions such as visual presentations of landmarks. As this analysis focused only on older adults, an (5) age limit of ≥ 60 years was set. No other inclusion or exclusion criteria were applied.

### Procedure

All participants underwent two assessment sessions, scheduled in the morning and separated by a minimum of seven days. During session one, participants were individually guided through the various questionnaires within their own rooms. Questions were asked by the researchers and large-print answer aids were provided to facilitate residents’ answering. If possible, the Depression in Age Scale (DIAS) was self-admistered by the participants to protect participants privacy. The second assessment, a measurement circuit for physical status and spatial orientation, took place in one of the nursing home’s open areas.

### Measurements

#### Socio-demographic and medical data

The socio-demographic and medical data (gender, age, care level) were provided by nursing home staff. The five care levels, a standardised classification in the German healthcare system, express a person’s degree of independence or impairment in daily life. People with care level 1 are defined as those with minor impairment of independence or abilities. People with care level 5 have severe impairment of independence or abilities with special requirements for nursing care [24]. All measures were performed following a standardized manual. More detailed information on the individual measures can be found in the study protocol in the section ‘Outcome measures’ [23].

#### Physical activity

PA was operationalized as steps per day, measured using the ‘activPAL4™ micro’ accelerometer (PAL Technologies Ltd., Glasgow, Scotland). The accelerometer was attached to the participants’ anterior mid-thigh and worn continuously for at least eight consecutive days. The day of attachment and removal were excluded from the analysis, ensuring a minimum of six full days of measurement. Raw data were analyzed using software (PALanalysis) provided by the developer.

#### Physical capacity

PC was measured using the habitual gait speed, which was determined using a 4-m walk [6, 25].

#### Objective motor capacity

Objective motor capacity was measured using the Short Physical Performance Battery (SPPB), a standardized instrument to assess lower extremity function [26], the timed up-and-go test (TUG) [27], and hand grip strength (Jamar^®^ hydraulic hand dynamometer, Model 5030J1, J.A. Preston Corporation, Clifton, NJ) [28].

#### Proxy-rated functional performance

Proxy-rated functional performance measures included the Barthel Index [29], an observer-based measure of independence in performing ADLs, and the Nursing Home Life Space Diameter (NHLSD) [30], for which nursing staff rate subjects’ life space mobility in four life spaces: (1) the resident’s private room (2), the area within the care unit (3), the area within the facility but outside the care unit, and (4) the area outside the facility grounds.

#### Cognitive performance

Cognitive performance was assessed using the Montreal Cognitive Assessment (MoCa), a 10-minute screening tool to detect mild cognitive impairment (MCI) [31].

#### Spatial orientation

Spatial orientation was assessed using two facility-specific tests developed and utilized in a previous feasibility study [23, 32]. The Landmark Recognition Test (LRT) employs a set of twelve photos (15 × 20 cm) comprising eight familiar landmarks from the residents’ everyday environment (two per life-space area) and four unfamiliar landmarks (one per life-space area) taken from another facility. Participants need to identify which landmarks can or cannot be found in or around the facility.

For the Landmark Sequence Test, another spatial orientation test, the eight landmark photos within and around the facility are presented in a standardized-randomized order. Participants were instructed to arrange the pictures in the order they would encounter the depicted locations when leaving their room. Participants receive one point for each correctly selected photo corresponding to the respective life space. The German questionnaire ‘Fragebogen Räumliche Strategien’ (FRS) assesses self-reported spatial cognition in individuals [33]. Its 19 items, measuring global/egocentric orientation, overview, and cardinal direction, were specifically adapted to the target group.

#### Subjective mobility-related concerns

Subjective mobility-related concerns were rated conducting the German version of the Spatial Anxiety Scale (SAS) [34], which evaluates anxiety levels in eight situations involving spatial orientation skills, and the Short Falls Efficacy Scale - International (Short FES-I) Questionnaire [35], a measure of self-efficacy related to falls in older adults.

#### Psychosocial well-being

Psychosocial well-being was assessed applying the Depression in Age Scale (DIAS) [36], a valid questionnaire [37] consisting of ten items that assess psychological status over the past two weeks, and the Satisfaction with Life Scale (SWLS), a valid tool to measure individuals’ subjective life satisfaction in various ages [38, 39].

### Data analyses

In accordance with Koolen et al. [21], participants were divided into four subgroups based on their PC (gait speed, m/s) and PA (average steps per day, steps). A gait speed of < 0.5 m/s is linked to an increased risk for adverse events and mortality and therefore suggested as threshold for the very old individuals [40, 41]. Thus, high PC was defined as gait speed ≥ 0.5 m/s and low PC was defined as a walking < 0.5 m/s. High PA was defined as ≥ 2,500 average steps per day, based on Tudor-Locke et al. [42, 43], who suggest this as a cut-off for ‘Basal activity’ in older adults. Less than 2,500 average steps per day were defined as low PA. Based on these thresholds, participants were binarily distributed as follows: (Q1) ‘Can do – do do’: high PC, high PA; (Q2) ‘Can do –don’t do’: high PC, low PA; (Q3) ‘Can’t do – do do’: low PC and high PA; and (Q4) ‘Can’t do – don’t do’: low PC, low PA.

Normal distribution was checked for all scores within each quadrant and confirmed using the Kolmogorov-Smirnov test. Group differences were analyzed using the Kruskal-Wallis H test or Fisher’s exact test, depending on data distribution. *P*-values were corrected with the Bonferroni test. If those tests showed significant results for one variable, individual group differences and effect sizes were calculated. Effect sizes were calculated by dividing the *z*-score of the Mann-Whitney U test by the square root of the number of the sample *n*: [*r* = *z*/√ (*n*)] [44]. Effect sizes of 0.1 - <0.3 are low, 0.3 - <0.5 are moderate, and equal or greater 0.5 are large. Correlation between average steps per day and gait speed were calculated using the Spearman rank correlation coefficient. Statistical analyses were performed using SPSS 27.0. (IBM Corp., Armonk, NY, USA) and Excel 2016 (Microsoft, Seattle, WA, USA).

## Results

**Fig. 1:**
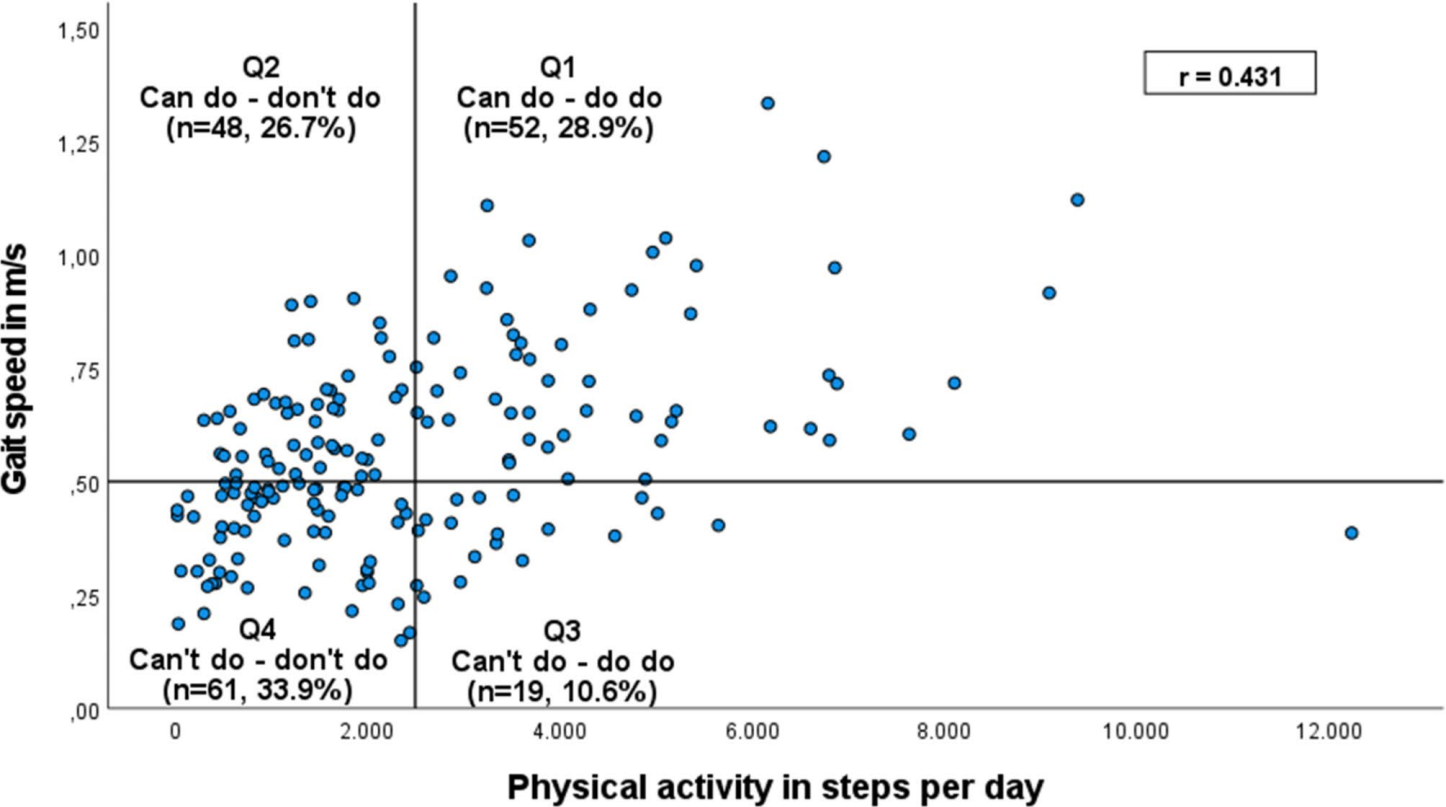
Group distribution based on the PC–PA quadrant concept. Cut-off for gait speed: 0.5 m/s. Cut-off for PA: 2,500 average steps per day

A total of 180 nursing home residents were included in the analysis. The PC-PA group distribution resulted in the following sample sizes per quadrant: (Q1) *n* = 52 (41 females), (Q2) *n* = 48 (34 females), (Q3) *n* = 19 (17 females), (Q4) *n* = 61 (51 females) (Fig. 1). Group characteristics and significance level of group differences are displayed in Additional file 1.

### Group comparison of significantly different characteristics

**Fig. 2:**
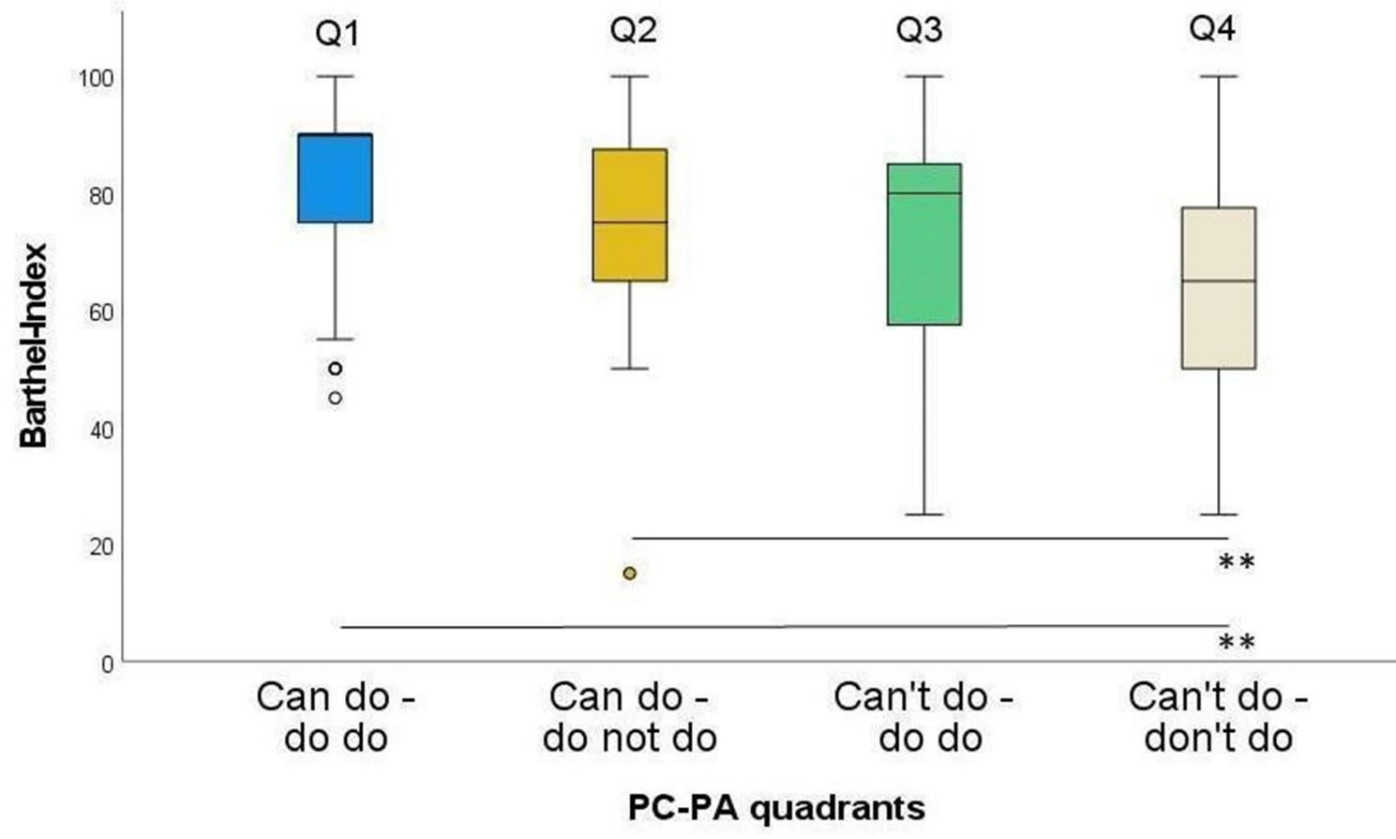
Barthel-Index in the four PC-PA groups. * *p* < 0.05; ** *p* < 0.01

The Barthel-Index differed significantly between Q1 and Q2, Q1 and Q4 as well as Q2 and Q4 (Fig. 2). Strongest effect sizes were found comparing Q1 and Q4 (ES = 0.57), while the weakest ES was found for the difference between Q2 and Q3 (ES = 0.05) (Table 1).

**Table 1:** Barthel-Index by quadrant group

Groups	*n*	Z	Sig.	Adj. Sig.^a^	ES
Q1-Q2	100	2.63	0.009	0.051	0.26
Q1-Q3	72	2.42	0.015	0.092	0.29
Q1-Q4	116	6.17	< 0.001	< 0.001**	0.57
Q2-Q3	68	0.45	0.652	1.000	0.05
Q2-Q4	112	3.34	< 0.001	0.005**	0.32
Q3-Q4	84	Q2	0.045	0.272	0.22

ES: effect size; Q1: Can do – do do, Q2: Can do – don’t do, Q3: Can’t – do do, Q4: Can’t – don’t do. ^a^significance values adjusted by the Bonferroni correction for multiple testing. * *p* < 0.05; ** *p* < 0.01

**Fig. 3:**
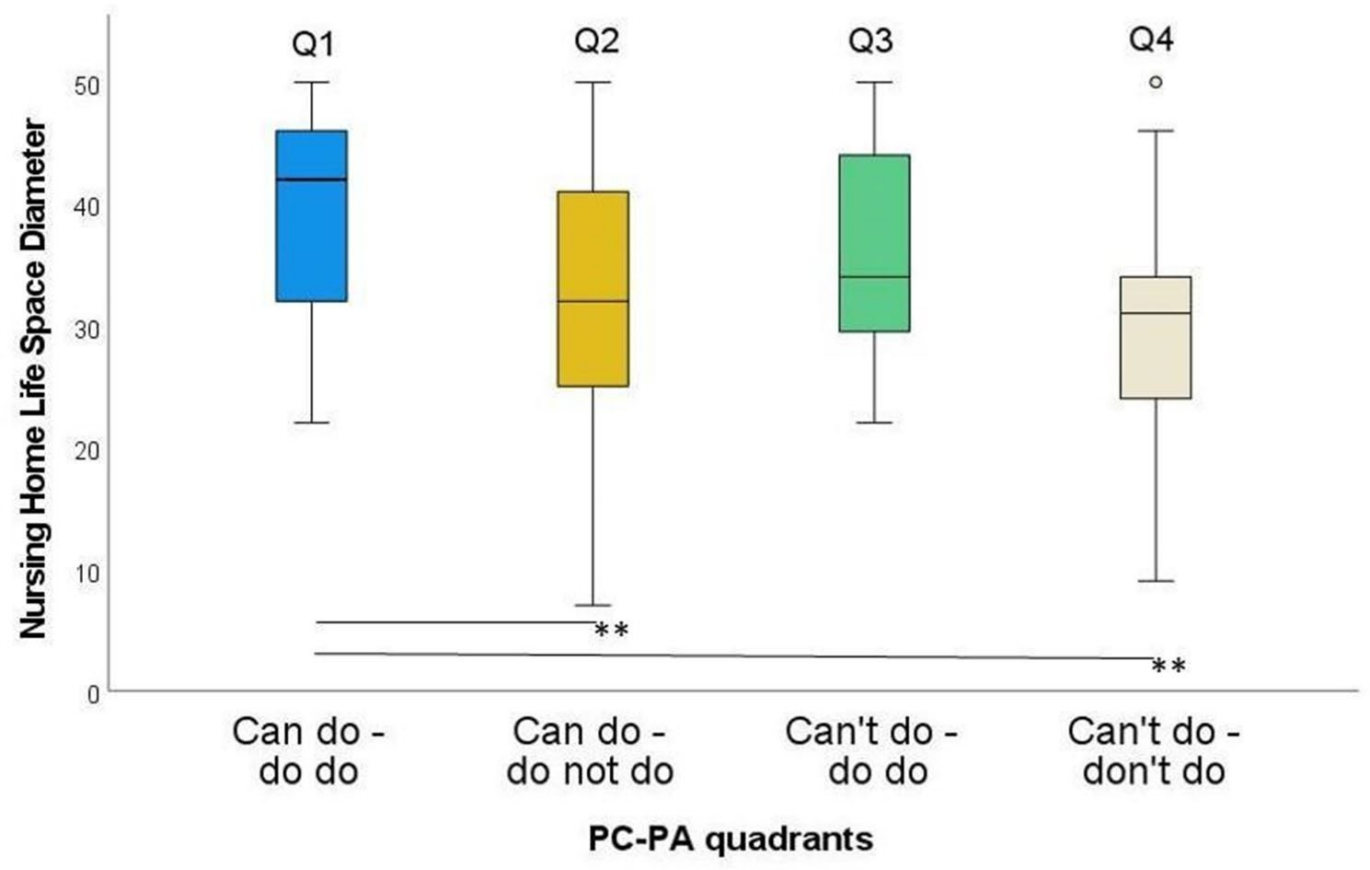
NHLSD in the four PC-PA groups. * *p* < 0.05; ** *p* < 0.01

In the NHLSD, Q1 differed significantly from Q2 and Q4 (Fig. 3). The largest difference was found between Q1 and Q4 (ES = 0.48), the lowest difference between Q2 and Q4 (ES = 0.14) (Table 2).

**Table 2:** Nursing Home Life Space Diameter (NHLSD) by quadrant group

Groups	*n*	Z	Sig.	Adj. Sig.^a^	ES
Q1-Q2	99	3.51	< 0.001	0.003**	0.35
Q1-Q3	72	1.35	0.178	1.000	0.16
Q1-Q4	117	5.20	< 0.001	< 0.001**	0.48
Q2-Q3	67	-1.28	0.201	1.000	0.16
Q2-Q4	112	1.44	0.150	0.901	0.14
Q3-Q4	85	2.39	0.017	0.102	0.26

ES: effect size; Q1: Can do – do do, Q2: Can do – don’t do, Q3: Can’t – do do, Q4: Can’t – don’t do. ^a^significance values adjusted by the Bonferroni correction for multiple testing. * *p* < 0.05; ** *p* < 0.01

**Fig. 4:**
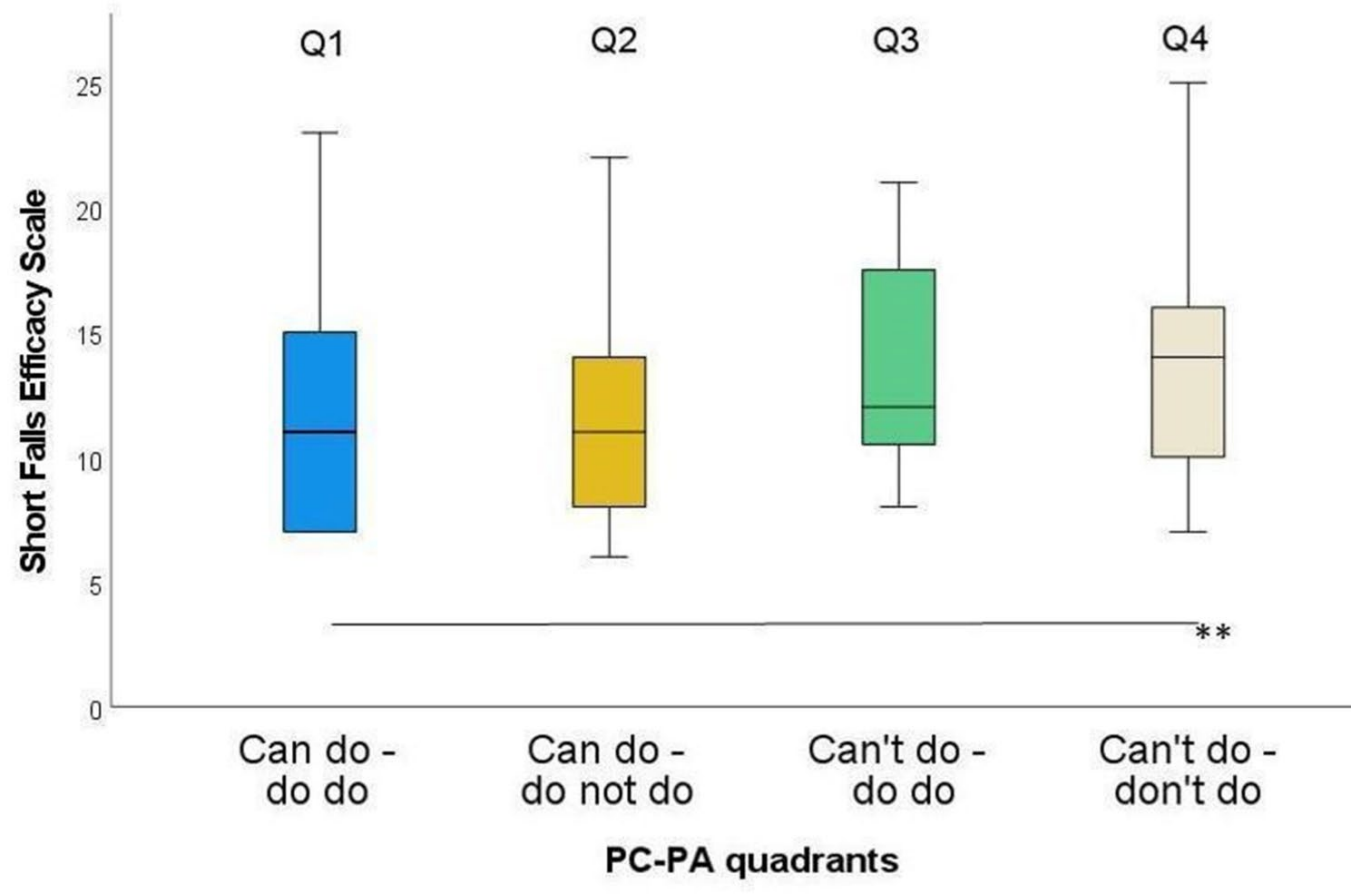
Short FES-I in the four PC-PA groups. * *p* < 0.05; ** *p* < 0.01

For the Short FES-I, only Q1 and Q4 differed significantly (Fig. 4). The strongest ES was found comparing these groups (ES = 0.36), while the weakest ES appeared between Q3 and Q4 (ES < 0.01) (Table 3).

**Table 3:** Short Falls Efficacy Scale - International (short FES-I) by quadrant group

Groups	*n*	Z	Sig.	Adj. Sig.^a^	ES
Q1-Q2	52	-0.44	0.662	1.000	0.06
Q1-Q3	116	-2.29	0.022	0.134	0.21
Q1-Q4	72	-3.23	0.001	0.007**	0.38
Q2-Q3	106	-1.88	0.059	0.357	0.18
Q2-Q4	62	-2.60	0.009	0.057	0.33
Q3-Q4	84	-0.03	0.974	1.000	< 0.01

ES: effect size; Q1: Can do – do do, Q2: Can do – don’t do, Q3: Can’t – do do, Q4: Can’t – don’t do. ^a^significance values adjusted by the Bonferroni correction for multiple testing. * *p* < 0.05; ** *p* < 0.01

**Fig. 5:**
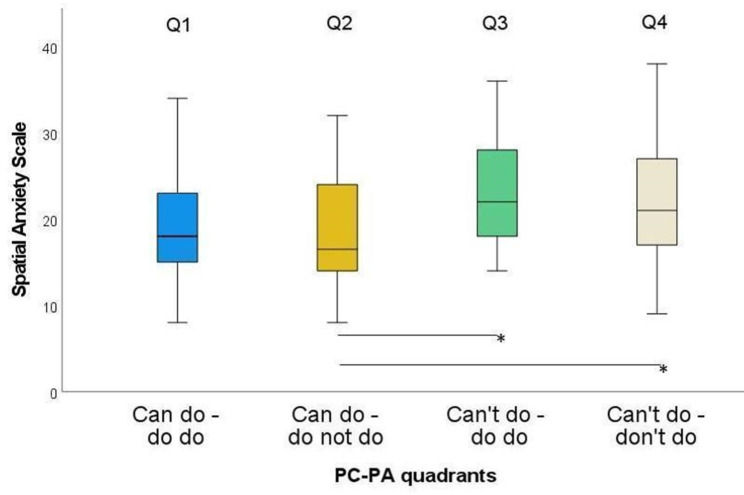
Spatial Anxiety Scale in the four PC-PA groups. * *p* < 0.05 ** *p* < 0.01

Considering the SAS, Q2 differed significantly to Q3 and Q4 (Fig. 5). The largest difference was found between Q2 and Q3 (ES = 0.35) and the smallest difference was found between Q1 and Q2 (ES = 0.07) (Table 4).

**Table 4:** Spatial anxiety scale by quadrant group

Groups	*n*	Z	Sig.	Adj. Sig.^a^	ES
Q1-Q2	89	0.66	0.511	1.000	0.07
Q1-Q3	65	-2.28	0.023	0.137	0.28
Q1-Q4	104	-2.03	0.043	0.257	0.20
Q2-Q3	62	-2.75	0.006	0.035*	0.35
Q2-Q4	101	-2.67	0.008	0.045*	0.27
Q4-Q3	77	0.83	0.406	1.000	0.09

ES: effect size; Q1: Can do – do do, Q2: Can do – don’t do, Q3: Can’t – do do, Q4: Can’t – don’t do. ^a^ significance values adjusted by the Bonferroni correction for multiple testing. * *p* < 0.05; ** *p* < 0.01

**Fig. 6:**
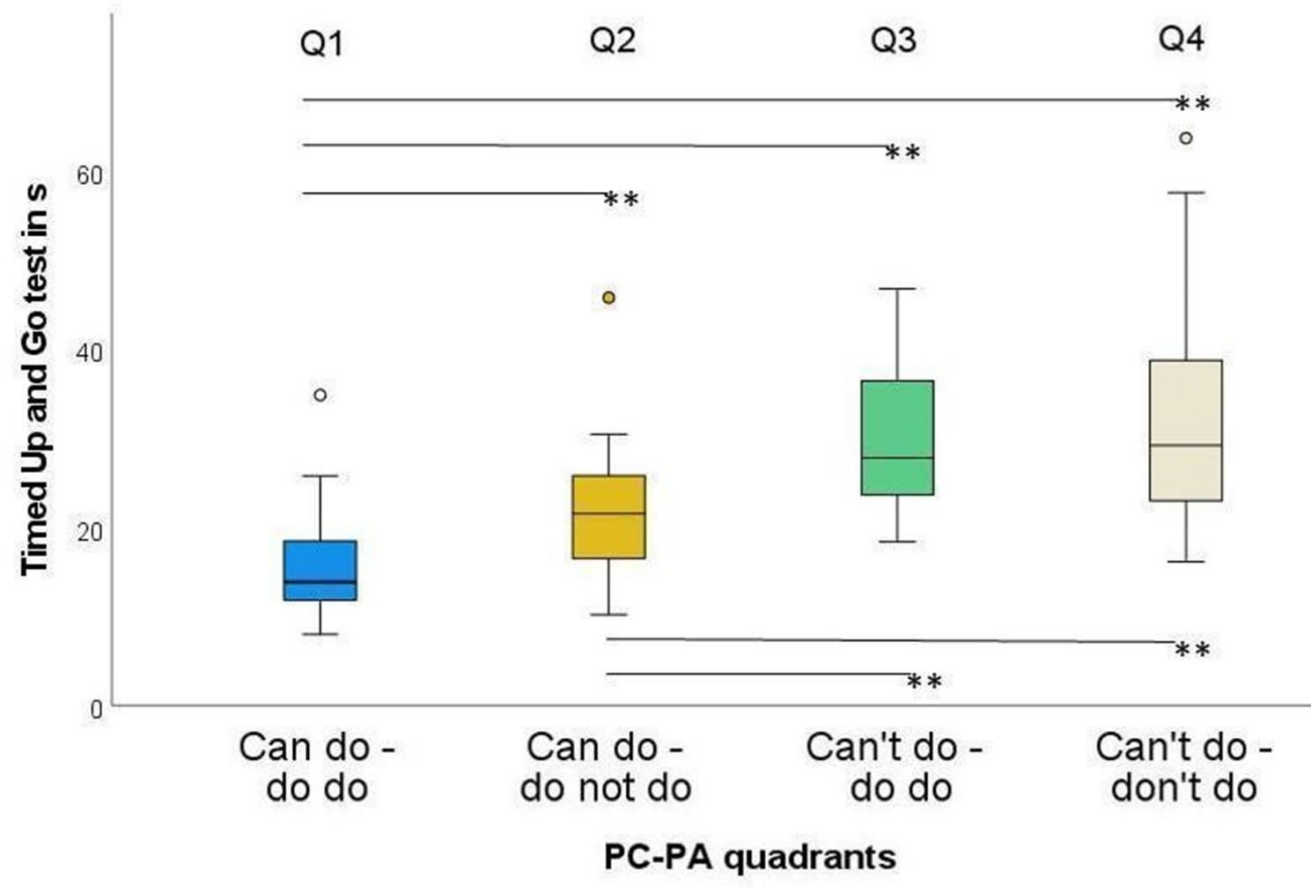
Timed up and go in the four PC-PA groups. * *p* < 0.05; ** *p* < 0.01

With respect to the TUG, all groups differed significantly except Q3 and Q4 (Fig. 6). ES varied from 0.01 to 0.99, while the strongest ES were found between Q1 and Q4 (Table 5).

**Table 5:** Timed up and go test by quadrant group

Groups	*n*	Z	Sig.	Adj. Sig.^a^	ES
Q1-Q2	101	-3.62	< 0.001	0.002**	0.36
Q1-Q3	115	-5.94	< 0.001	< 0.001**	0.55
Q1-Q4	73	-8.46	< 0.001	< 0.001**	0.99
Q2-Q3	110	-3.18	0.002	0.009**	0.30
Q2-Q4	68	-4.54	< 0.001	< 0.001**	0.55
Q3-Q4	82	-0.11	0.911	1.000	0.01

ES: effect size; Q1: Can do – do do, Q2: Can do – don’t do, Q3: Can’t – do do, Q4: Can’t – don’t do. ^a^significance values adjusted by the Bonferroni correction for multiple testing. * *p* < 0.05; ** *p* < 0.01

**Fig. 7:**
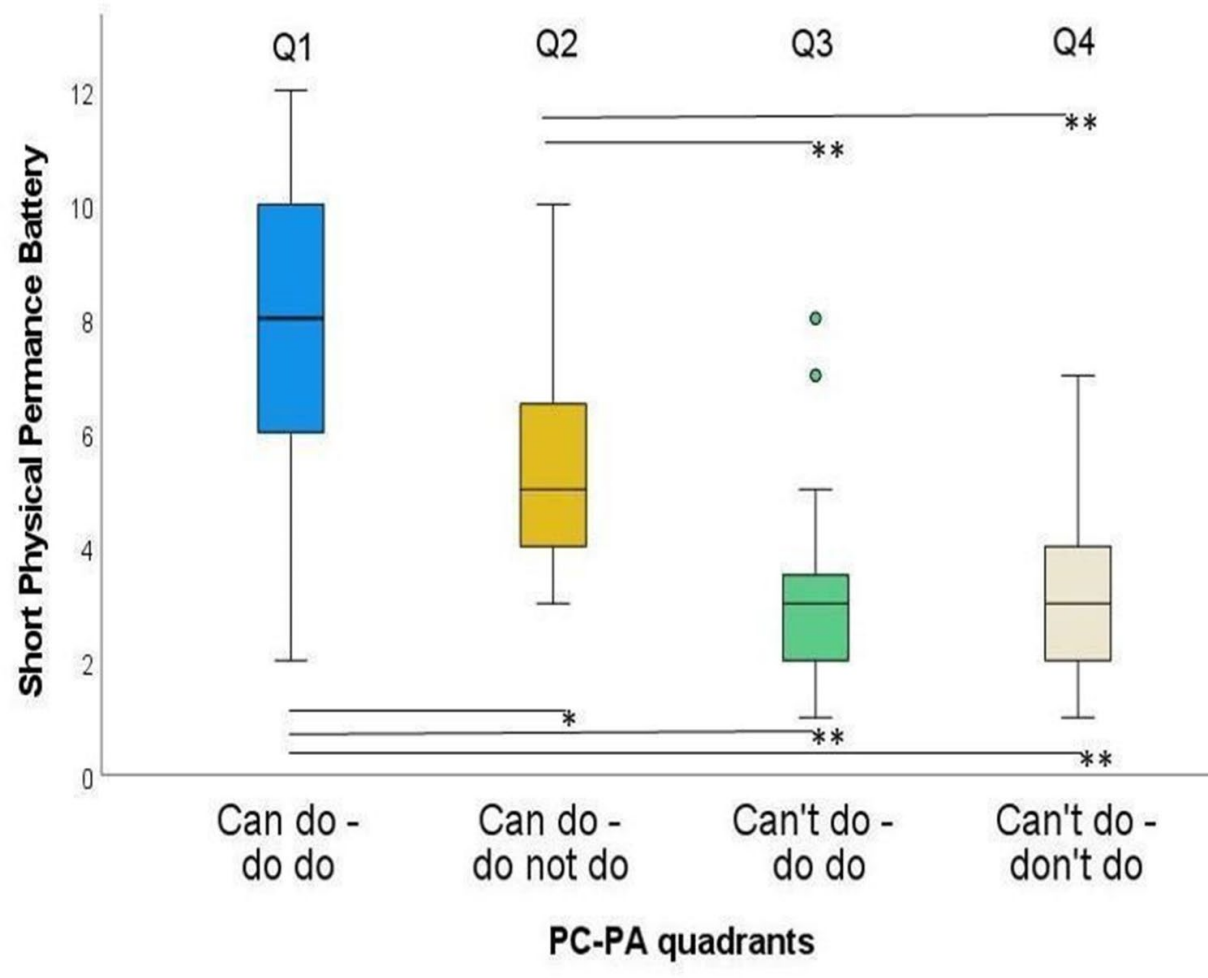
Short Physical Performance Battery in the four PC-PA groups. * *p* < 0.05; ** *p* < 0.01

The SPPB presented significant differences between all four groups except for Q3 and Q4 (Fig. 7). ES ranged from 0.02 to 1.10, while the largest difference was found between Q1 and Q3 (Table 6).

**Table 6:** Short physical performance battery by quadrant group

Groups	*n*	Z	Sig.	Adj. Sig.^a^	ES
Q1-Q2	42	3.16	< 0.002	0.010*	0.49
Q1-Q3	30	6.00	< 0.001	< 0.001**	1.10
Q1-Q4	64	8.62	< 0.001	< 0.001**	1.08
Q2-Q3	30	3.60	0.003	0.002**	0.66
Q2-Q4	64	5.19	< 0.001	< 0.001**	0.65
Q3-Q4	52	0.12	0.904	1.000	0.02

ES: effect size; Q1: Can do – do do, Q2: Can do – don’t do, Q3: Can’t – do do, Q4: Can’t – don’t do. ^a^significance values adjusted by the Bonferroni correction for multiple testing. * *p* < 0.05; ** *p* < 0.01

## Discussion

This study aimed to identify contextual factors in nursing home residents with aligned or misaligned physical activity and physical capacity.

To the best of our knowledge, this study applied the PC-PA concept for the first time in nursing home residents. The first hypothesis about the superiority of Q1 vs. Q4 for all variables measured in this study was only partly confirmed. As expected, we found significantly greater objective motor capacity, proxy-rated functional performance and lower fear of falling for Q1 than for Q4. Against our expectations, our results show that Q1 was not superior concerning cognitive performance, orientation, psycho-social well-being, and spatial anxiety, suggesting that these variables do not discriminate between those with high PA/PC and those with low PA/PC. Regarding our second hypothesis, we found that Q1 presents significantly higher objective motor capacity and life-space mobility than Q2. Noteworthy, no variable discriminated between Q3 and Q4.

### Group distribution to the PC-PA concept

29% of the sample were part of Q1, with aligned high PA (4,067 median steps per day) and PC (median gait speed of 0.7 m/s), compared to other long-term care samples [1, 3]. Still, PA in this subgroup is below the 5,000-step threshold defined as ‘limited activity’ by Tudor-Locke et al. [42, 43]; gait speed is below the frailty level of 0.8 m/s [45, 46].

Approximately one-third were in Q4, with aligned very low PC (median gait speed 0.4 m/s) and PA (median 971 steps per day). Gait speed in this group is distinctly lower than in geriatric hospital settings (0.58 m/s) and acute care settings (0.46 m/s) [47], underlining this group’s low health status.

27% were in the misaligned group that could potentially be physically active based on their PC (median gait speed: 0.6 m/s), but their PA level is low (i.e., median steps 1,437). Reasons for this discrepancy remain unclear to this point.

The counterpart to this group is about 10% of the residents who are physically active (median steps per day: 3,343) despite a low PC (median gait speed: 0.4 m/s). This misaligned group (Q3) reaches a surprisingly high PA level for reasons yet to be identified. In addition, there is likely an increased risk for falls in this group due to their low physical function [48] but increased exposure.

### H1: can do - do do (Q1) vs. can’t do - don’t do (Q4)

As hypothesized, Q1 is significantly more independent in ADL compared to the Q4 group (ES: 0.57; Table 1). This is also the case for Q2, suggesting that limitations in ADL are more strongly related to low PC than to PA. Since we used gait speed as a measure of PC in this study, our results suggest that gait speed is an important factor in reducing nursing staff burden and should definitely be specifically addressed in long-term care. This is in line with a study by Idland et al., in which gait speed was the strongest predictor for onset of disability in ADL in nursing home residents [49].

Considerably higher PA in Q1 compared to the Q4 is reflected in a significantly more frequent use of different life spaces (ES: 0.48; Table 2). This is consistent with our expectations based on previous studies, who found positive associations between nursing home residents’ life-space mobility and their physical performance [50–52]. For example, Sverdrup et al. [51] found that better physical performance in nursing home residents is associated with a wider life-space. These studies also emphazise the importance of mobility in the living environment for cognition and psychosocial well-being [50–52]. Thus, promoting life-space exploration is a promising approach when planning activities to increase PA and health in nursing homes.

As expected, fear of falling was significantly higher in Q4 compared to Q1 (ES: 0.38; Table 3). This is in line with previous studies demonstrating that mobility-related concerns are associated with poorer physical performance [53] and PA [54–56]. Mobility-related concerns should therefore be considered when planning interventions for the Q4 group.

The strongest differences between Q1 and Q4 occurred in objective motor performance measures (ES: 0.99–1.08; Tables 5 and 6). This of course is trivial considering PC being the separating factor between those two groups. However, as this factor is the strongest of all, it becomes clear that objective motor capacity is a key factor for PA in nursing home residents. This is consistent with the results of a systematic review by Jansen et al. [57], who found 6 studies that provided evidence for the positive effects of exercise on physical activity in nursing home residents. Therefore, exercise should be considered when planning interventions.

Unexpectedly, no differences appeared when comparing cognitive performance, orientation, and spatial anxiety between the Q4 and Q1, since previous studies indicate correlations between those factors and both PC and PA [3, 56, 58, 59]. In contrast, this study indicates that different levels of PA and PC are independent of their cognitive status or orientation skills. One reason for this may be that people with low cognitive status also tend to wander as a symptom associated with dementia [60]. Also, social support and environmental construction might play an important role in the PA behavior of nursing home residents [61, 62]. Another reason might be our inclusion criteria, which demand the ability to participate in group activities. This led to the exclusion of many residents with severe dementia, limiting our results to residents with mild to moderate dementia. Nonetheless, the fact that cognition appears to have no impact on PA in this population warrants further research.

Moreover, Q1 and Q4 did not differ regarding psychosocial wellbeing. This contradicts our expectations, based on previous studies showing associations of PA with quality of life and depression in older adults [3, 4]. This might be related to high levels of PA in our study compared to other studies. Despite similar or even more strict inclusion criteria, Arrieta et al. [3] recorded an average number of steps which is comparable to our two groups with low PA (Q2 and Q4). The average PA in our study was considerably higher and depressive symptoms may be more prevalent in populations with lower PA. Nevertheless, psychosocial wellbeing is a complex construct that is difficult to measure, especially in a heterogeneous and special setting such as nursing homes. For example, a person with a high cognitive and physical status may enjoy a safe and active life in a nursing home, while at the same time being very frustrated by the environment and loss of independence. A person with low PA might be depressive or enjoying a carefree last episode of life. It is therefore reasonable that psychosocial wellbeing did not differ across the four groups. Further research is needed to better understand the reasons for low psychosocial wellbeing in nursing home residents.

### H2: Can do - Do do (Q1) vs. Can do - Don’t do (Q2)

A specific goal of this study was to understand reasons for misalignment between PA and PC in nursing home residents. Comparing Q1 and Q2 we found a considerably lower number of steps in the latter, despite a median gait speed of 0.6 (IQR: 0.1) m/s above the average for nursing home residents [40]. Interestingly, we also found a significantly higher life-space mobility for the first one (ES: 0.35; Table 2). This means that higher PA levels in Q1 are not only generated by making more steps but moving more frequently in different life spaces. Therefore, the individually perceived attraction to visit different life spaces might be critical to understanding why Q2 has such a low PA and is a modifiable factor in nursing homes.

We also found significant differences between the groups regarding objective motor capacity, despite gait speed being similar in both groups (Tables 5 and 6). Although it is important to see that PA behavior is multifactorial and objective motor capacity is only one aspect, our results indicate that there might be thresholds of specific motor functions, such as reflected in the TUG and SPPB, leading to higher or lower PA in nursing home residents despite a normal gait speed of at least 0.5 m/s. Even in this group of relatively fit older people (Q2), it could be perceived as much more strenuous to walk more and/or visit other living areas if certain motor skills are less developed. Future studies need to prove this hypothesis and determine those critical thresholds for various motor skills to train those in the Q2.

Our measures of psychosocial well-being, orientation, cognition, and subjective mobility-related concerns do not explain group differences between the ‘Can do’ groups and the misalignment of PC and PA in Q2. There are likely other influential factors not measured in this study. For example, one other key factor for PA in nursing home residents is motivation [63]. Promising interventions increasing motivation for exercising and PA in long-term care residents have been published previously [64, 65]. Future studies need to determine the role of motivation for PA in the Q2. For this purpose, the PC-PA concept can be an explicitly useful tool, as presented in this study.

### H2: can’t do - do do (Q3) vs. can’t do - don’t do (Q4)

Surprisingly, none of the variables we tested in this study differed between Q3 and Q4. In contrast to the ‘Can do’ groups, also life-space mobility and motor capacity appear to have no impact on different PA levels in the ‘Can’t do’ groups. The role of motor capacity in PA behavior might therefore be dependent on a basic level of normal gait speed above 0.5 m/s. Below this level, other variables might be more relevant. However, this study was not able to explain differences in PA behavior despite a low PC and identify underlying reasons for misaligned PC and PA in Q3. This raises further questions on how PA behavior comes about. It remains unclear why those in Q3 take a median of more than 3,300 steps per day.

Hand grip strength did not differ between any group in this study. Grip strength is a powerful predictor of health-related outcomes, simple and quick to use, and recommended as a first-line screening tool for sarcopenia [66]. However, associations with lower limb muscle function are weak and results inconsistent [67–69]. Based on our results and in line with previous studies, we recommend not to use hand grip as a screening tool for lower limb function in nursing home residents and use specific tests instead.

### Limitations

The operationalization of PA in this study by the average number of steps per day might not provide a complete picture. Activities such as group exercises in seated positions are often conducted in nursing homes and are not recorded by the movement sensor. This may have led to an underestimation of PA in some participants who move their upper limbs a lot, and they may have been misclassified as less physically active. To the best of our knowledge, this is the first study measuring spatial orientation skills in nursing home residents. Therefore, we recognize that measures for spatial orientation might need further adjustment to assess nursing home residents’ specific needs for navigation and be able to detect significant differences between subgroups of nursing home residents. Despite the considerable size of our study sample in this challenging population, the number of participants per nursing home were too diverse and in some cases too small to carry out a meaningful statistical analysis. Therefore, we cannot rule out the possibility that the statements only apply to participants from certain nursing homes. Finally, our results are linked to the cut-off values we used. The application of different cut-off values could lead to different group distributions and other results.

### Impact for future studies

The variables found in this study may support future efforts to plan specific, targeted interventions for improving PA in nursing home residents. Future studies may also consider and investigate variables such as social support, motivation, cognition, self-efficacy to be physically active, nursing home organization and environmental factors influencing PA. Another unclear point from these findings is why the quality of life is perceived to be poor in nursing homes and whether and how this is related to PA. Regarding the relationship between PC and PA, future studies could try to find out if there is a threshold of PC that determines whether people in nursing homes can be physically active or not. The measurement of spatial orientation could be improved by digitalizing measurement methods, for example through instrumented digital applications or virtual reality, where landmarks of nursing homes could be reconstructed virtually.

## Conclusion

There are specific contextual factors that are able to distinguish between nursing home residents when these are subdivided into mutually exclusive PC-PA quadrants. Some (i.e., objective motor capacity, life-space mobility, ADL, and subjective mobility related concerns) but not all (i.e., cognition, orientation, and psychosocial well-being) of the expected variables discriminated the subgroups from each other.

## Electronic supplementary material

Below is the link to the electronic supplementary material.

Supplementary Material 1

## Data Availability

The datasets used and/or analyzed during the current study are available from the corresponding author on reasonable request.

## Abbreviations

ADL: Activities of daily living
DIA: Depression in Age Scale
ES: Effect sizes
MOCA: Montreal Cognitive Assessment
NHLSD: Nursing home life-space mobility
PA: Physical activity
PC: Physical capacity
SAS: Spatial Anxiety Scale
Short FES: I-Short Falls Efficacy Scale-International
SPPB: Short Physical Performance Battery
SWLS: Satisfaction with Life Scale
TUG: Timed up and go test
