# Supplementary material for: ‘Can do’ versus ‘Do do’ in nursing home residents: identification of contextual factors discriminating groups with aligned or misaligned physical activity and physical capacity
Source: Eur Rev Aging Phys Act. 2024 Nov 12;21:30. doi: 10.1186/s11556-024-00365-4 (PMC11558847; doi:10.1186/s11556-024-00365-4)
Supplement: Supplementary file 1 — Supplementary Material 1 [file 11556_2024_365_MOESM1_ESM.docx]

**Additional file 1**

Descriptive characteristics of the four PA–PC quadrants and significance level of group differences

|  | **Can** **do**–  **do** **do (Q1)** | | **Can** **do**–  **don’t** **do (Q2)** | | **Can’t** **do**–  **do** **do (Q3)** | | **Can’t** **do**–  **don’t** **do (Q4)** | | ***p^a^*** |
| --- | --- | --- | --- | --- | --- | --- | --- | --- | --- |
|  | **Mean (SD)** | **Median (IQR)** | **Mean (SD)** | **Median (IQR)** | **Mean (SD)** | **Median (IQR)** | **Mean (SD)** | **Median (IQR)** |  |
| ***Descriptives*** |  |  |  |  |  |  |  |  |  |
| *N* | 52 | - | 48 | - | 19 | - | 61 | - | - |
| *Female [%]* | 41 [78.8] | - | 34 [70.8] | - | 17 [89.5] | - | 51 [83.6] | - | 0.28^b^ |
| *Age, years* | 81.2 (8.4) | 82 (13) | 84.4 (8.6) | 84 (1) | 83.6 (9.1) | 84 (12) | 84.8 (7.2) | 85 (8) | 0.12 |
| *BMI, kg/m²* | 26.7 (4.2) | 26.1 (5) | 27.1 (6.7) | 27.4 (6.9) | 29.1 (6.1) | 29.1 (5.8) | 28.8 (5.9) | 27.6 (7.7) | 0.13 |
| *Care level, 1* – *5 p.* | 2.7 (0.6) | 3 (1) | 2.8 (0.8) | 3 (1) | 2.8 (0.7) | 3 (1) | 2.9 (0.7) | 3 (1) | 0.38 |
| ***Physical activity*** |  |  |  |  |  |  |  |  |  |
| *Steps per day, steps* | 4651 (1704) | 4067 (1935) | 1397 (544) | 1438 (810) | 3968 (2201) | 3343 (1703) | 1108 (720) | 971 (1254) | <0.01** |
| ***Physical capacity*** |  |  |  |  |  |  |  |  |  |
| *Walking speed, m/s* | 0.8 (0.2) | 0.7 (0.3) | 0.6 (0.1) | 0.6 (0.14) | 0.4 (0.1) | 0.4 (0.1) | 0.4 (0.1) | 0.4 (0.2) | <0.01** |
| ***Objective motor capacity*** |  |  |  |  |  |  |  |  |  |
| *TUG, s* | 15.7 (5.5) | 13.8 (6.7) | 21.4 (6.6) | 21.5 (10) | 30.7 (9.1) | 27.8 (14.2) | 32.0 (11.2) | 29.2 (16.9) | <0.01** |
| *SPPB, 0-12 p.* | 7.7 (2.5) | 8 (4) | 5.5 (1.9) | 5 (2.8) | 3.3 (1.9) | 3 (2) | 3.1 (1.5) | 3 (2) | <0.01** |
| *Hand grip strength, kg* | 20.5 (6.9) | 20 (8) | 18.6 (7.3) | 16.9 (6.6) | 17.5 (7.4) | 16 (9.7) | 18.1 (6.7) | 16.2 (8.6) | 0.12 |
| ***Proxy-rated functional performance*** |  |  |  |  |  |  |  |  |  |
| *Barthel Index, 0* – *100 p.* | 83.6 (14.9) | 90 (15) | 75.1 (15.7) | 75 (24) | 71.3 (21.7) | 80 (30) | 63.4 (16.6) | 65 (30) | <0.01** |
| *NHLSD, 0* – *50 p.* | 39.6 (8.5) | 42 (14) | 32.4 (11.0) | 32 (17) | 36.2 (9.2) | 34 (18) | 29.5 (8.2) | 31 (10) | <0.01** |
| ***Cognition*** |  |  |  |  |  |  |  |  |  |
| *MoCA* – *total* | 17.7 (5.0) | 17 (8) | 16.3 (5.4) | 17 (9) | 16.1 (5.4) | 16 (7) | 16.8 (5.9) | 17 (11) | 0.65 |
| ***Spatial Orientation*** |  |  |  |  |  |  |  |  |  |
| *LM recognition* | 0.6 (0.2) | 0.6 (0.3) | 0.6 (0.3) | 0.6 (0.5) | 0.6 (0.3) | 0.5 (0.5) | 0.5 (0.3) | 0.5 (0.3) | 0.81 |
| *LM sequence* | 2.6 (13.6) | 0.8 (0.4) | 0.8 (0.2) | 0.8 (0.4) | 0.8 (0.2) | 0.8 (0.4) | 0.7 (0.3) | 0.8 (0.4) | 0.82 |
| *FRS – Cardinal direction* | 4.3 (1.7) | 4.5 (2.8) | 4.8 (2.0) | 5.5 (3.8) | 4.1 (1.8) | 4 (2.8) | 3.9 (1.9) | 3.5 (3.5) | 0.13 |
| *FRS – Overview* | 4.2 (1.3) | 4.1 (2) | 4.1 (1.4) | 4.3 (2.4) | 4.3 (1.2) | 4.3 (2.1) | 4.1 (1.1) | 4.1 (1.4) | 0.96 |
| *FRS - Orientation global/egocentric* | 4.9 (1.1) | 5 (1.9) | 5.0 (1.1) | 5 (1.4) | 4.8 (0.9) | 4.8 (1.2) | 4.7 (1.2) | 5.1 (1.3) | 0.63 |
| **Subjective mobility-related concerns** |  |  |  |  |  |  |  |  |  |
| *Fear of falling,  Short FES-I* | 11.2 (4.4) | 11 (8) | 11.5 (4.2) | 11 (7) | 13.8 (4.4) | 12 (8) | 14.0 (4.7) | 14 (6) | <0.01** |
| *Spatial anxiety, SAS* | 19.2 (6.4) | 18 (9) | 18.1 (6.6) | 16.5 (11) | 23.6 (6.3) | 22 (11) | 22.0 (6.3) | 21 (10) | 0.01* |
| ***Psychosocial well-being*** |  |  |  |  |  |  |  |  |  |
| *Life satisfaction, SWLS* | 14.7 (5.7) | 14 (7) | 15.7 (6.3) | 14.5 (8) | 15.1 (6.9) | 14 (11) | 16.2 (5.9) | 16 (9) | 0.50 |
| *Depression, DIA* | 2.9 (2.5) | 3 (3) | 3.8 (2.9) | 4 (4) | 3.5 (2.7) | 3 (6) | 3.6 (2.7) | 4 (5) | 0.42 |

^a^ Calculated using Kruskal–Wallis H test; ^b^ Calculated using Fisher’s exact test; * *p*<0.05; ** *p*<0.01. BMI: body mass index; TUG: Timed Up and Go Test; SPPB: Short Physical Performance Battery; LM: Landmark; FRS: Fragebogen Räumliche Strategien; SWLS: Satisfaction with Life Scale; DIA: Depression in Age; Short FES-I: Short Falls Efficacy Scale - International; SAS: Spatial Anxiety Scale
